# A Predictive Clinical-Radiomics Nomogram for Survival Prediction of Glioblastoma Using MRI

**DOI:** 10.3390/diagnostics11112043

**Published:** 2021-11-04

**Authors:** Samy Ammari, Raoul Sallé de Chou, Corinne Balleyguier, Emilie Chouzenoux, Mehdi Touat, Arnaud Quillent, Sarah Dumont, Sophie Bockel, Gabriel C. T. E. Garcia, Mickael Elhaik, Bidault Francois, Valentin Borget, Nathalie Lassau, Mohamed Khettab, Tarek Assi

**Affiliations:** 1 Biomaps, UMR1281 INSERM, CEA, CNRS, Université Paris-Saclay, 94805 Villejuif, France; Samy.AMMARI@gustaveroussy.fr (S.A.); Corinne.BALLEYGUIER@gustaveroussy.fr (C.B.); Mickael.ELHAIK@gustaveroussy.fr (M.E.); Francois.BIDAULT@gustaveroussy.fr (B.F.); valentinborget@gmail.com (V.B.); Nathalie.LASSAU@gustaveroussy.fr (N.L.); 2 Department of Imaging, Gustave Roussy, Université Paris Saclay, 94805 Villejuif, France; Gabriel.garcia@gustaveroussy.fr; 3 Centre de Vision Numérique, OPIS, CentraleSupélec, Inria, Université Paris-Saclay, 91190 Gif-sur-Yvette, France; emilie.chouzenoux@inria.fr (E.C.); arnaud.quillent@inria.fr (A.Q.); 4 Service de Neurologie 2-Mazarin, AP-HP Hôpitaux Universitaires La Pitié Salpêtrière-Charles Foix, 75013 Paris, France; mehdi.touat@aphp.fr; 5 Institut du Cerveau et de la Moelle Epinière, CNRS, UMR S 1127, Inserm, Sorbonne Université, 75013 Paris, France; 6 Department of oncology, Gustave Roussy, Université Paris Saclay, 94805 Villejuif, France; SARAH.DUMONT@gustaveroussy.fr (S.D.); TAREK.ASSI@gustaveroussy.fr (T.A.); 7 Department of Radiation Oncology, Gustave Roussy Cancer Campus, 94800 Villejuif, France; Sophie.BOCKEL@gustaveroussy.fr; 8 Medical Oncology Unit, CHU de La Réunion, Université de La Réunion, 97410 Saint Pierre, France; MOHAMED.KHETTAB@gustaveroussy.fr

**Keywords:** glioblastoma, biomarker, radiomics, machine learning

## Abstract

Glioblastoma (GBM) is the most common and aggressive primary brain tumor in adult patients with a median survival of around one year. Prediction of survival outcomes in GBM patients could represent a huge step in treatment personalization. The objective of this study was to develop machine learning (ML) algorithms for survival prediction of GBM patient. We identified a radiomic signature on a training-set composed of data from the 2019 BraTS challenge (210 patients) from MRI retrieved at diagnosis. Then, using this signature along with the age of the patients for training classification models, we obtained on test-sets AUCs of 0.85, 0.74 and 0.58 (0.92, 0.88 and 0.75 on the training-sets) for survival at 9-, 12- and 15-months, respectively. This signature was then validated on an independent cohort of 116 GBM patients with confirmed disease relapse for the prediction of patients surviving less or more than the median OS of 22 months. Our model insured an AUC of 0.71 (0.65 on train). The Kaplan–Meier method showed significant OS difference between groups (log-rank *p* = 0.05). These results suggest that radiomic signatures may improve survival outcome predictions in GBM thus creating a solid clinical tool for tailoring therapy in this population.

## 1. Introduction

Glioblastoma (GBM), the most common and aggressive primary brain tumor in adult patients, is associated with a dismal prognosis [1]. The standard of care for the initial management of GBM is based on the maximal safe resection of the tumor followed by concomitant chemoradiotherapy and adjuvant chemotherapy with temozolomide [2,3,4]; nevertheless, the median survival of GBM patients remains merely equal to 12 months [5,6]. Prediction of survival outcomes in GBM patients at early stages constitutes a major step in the optimization of treatment selection and personalization. At present, only several potential prognostic tumor-specific biomarkers are identified in GBM patients. However, only a handful of these biomarkers have a significant role in daily clinical practice as prognostic or predictive biomarkers, such as O^6^-methylguanine-DNA-methyltransferase (MGMT) promoter methylation status [7]. On the other hand, it is sometimes challenging to accurately retrieve complex molecular variables given the high intratumor heterogeneity of GBM [8,9,10,11], which suggests that additional phenotypic signatures may improve prognosis stratification and enable better predicting long-term survival. Therefore, additional phenotypic signatures are eagerly needed to identify a subgroup of patients with long- or short-term survival.

Magnetic resonance imaging (MRI) is the key radiological test used by neuro-oncologists for the evaluation and follow-up of brain tumors [12]. It has emerged as a powerful non-invasive exam for the classification of diseases and prediction of treatment outcomes. Recent developments in the field of radiology have encouraged researchers to use radiomics in various indications. Radiomics were applied to retrieve the information from pixels in MR images, which consist of the extraction of shape, texture, and voxel’s intensity from the segmented tumor on the images. A multitude of studies has proposed several models based on radiomics for classification [13], treatment outcomes [14,15], or OS prediction [16,17,18,19,20,21,22] in brain tumors. For instance, Pak et al. [16] used radiomics from pre-operative DCE MRI to stratify GBM patients into high-risk and low-risk in terms of survival. Moreover, Kickingereder [17] successfully designed a radiomics signature composed of eight features to classify GBM patients into low-, medium- and long-risk survival subgroups using pre-operative post-T1 weighted, T2 FLAIR, and T2 weighted images. In addition, Sanghani et al. [18] tended to classify GBM patients and their survival outcomes into three distinct subgroups, based on the 2018 Brain Tumor Segmentation (BraTS) challenge datasets, their machine learning method achieved an accuracy of 87.5%. These papers have provided a solid confirmation on the potential role of radiomics in predicting OS in GBM patients; nonetheless, they suffer from the lack of sufficient patient data and most importantly, radiomics signatures with ease of use in daily practice have not been validated in all these papers. To ensure the robustness of a potential radiomics signature, the evaluation of different datasets is eagerly needed.

To our knowledge, the robustness and generalization error of radiomics signatures have never been evaluated on different datasets. In this study, machine learning (ML) models for survival prediction of GBM patients from the training dataset of the 2019 BraTS challenge were developed. Based on these models, we identified a radiomic signature to validate a completely independent cohort of GBM patients.

## 2. Materials and Methods

### 2.1. Dataset

#### 2.1.1. BraTS 2019 Cohort

Data from the train dataset of the 2019 BraTS challenge [23,24,25] were retrieved to train a neural network for brain tumor segmentation, and to select a set of radiomics features to be evaluated on a distinct cohort for the survival prediction of GBM. The initial train dataset was composed of 259 patients with GBM and 76 patients with lower-grade gliomas (LGG) with a pathologically confirmed diagnosis. The OS of 210 GBM patients were available in the training data for the identification of a radiomics signature, to be used for survival analysis.

For every patient, the dataset contained: the pre- (T1) and post-contrast T1-weighted (T1ce), the T2-weighted and fluid attenuated inversion recovery 3D MR images along with one ground truth mask containing 3 segmentation labels, covering 3 different anatomical parts of the tumor (the necrotic (NCR) and non-enhancing (NET) tumor core, the peri-tumoral edema (ED) and the enhancing tumor (ET)).

#### 2.1.2. Validation Cohort

This cohort was composed of 116 patients with histologically confirmed GBM (based on the World Health Organization [WHO] classification of central nervous system tumors, Grade IV [26]). All the radiological data were retrospectively collected from baseline MRI (at diagnosis) performed at Gustave Roussy Cancer Campus (Villejuif, France) between 2006 and 2016. All included patients, with an age ranging between 18 and 80 years old, had confirmed disease relapse and then received bevacizumab for the treatment of recurrent GBM. Disease relapses occurred after initial management by surgery (when possible) (70% patients) followed by post-operative chemo-radiotherapy or by chemo-radiotherapy alone. This study was approved by the institutional review board as per RGPD provisions and was declared on the Health Data Hub site and the CNIL as per RGPD recommendations. Whenever feasible, patients were informed of their enrolment in the study. Patients’ characteristics are summarized in Table 1.

MR acquisitions were all performed on 2 imaging machines (MRI) from the same manufacturer (General Electric, Milwaukee, WI, USA): Optima MR450w 1.5T and Discovery MR750w 3T. MRI data included a post-contrast (gadoterate meglumine, Dotarem, Guerbet, Villepinte, France) three-dimensional T1-weighted Fast Spoiled Gradient Recalled (FSPGR) acquisition (post-contrast 3DT1), post-contrast 3DT1, and fat-suppressed FLAIR images. MR images were only used as inputs of the radiomics classifier. To ensure image quality, neuro-radiologists analyzed all the available imaging sequences. Table 2 details the MRI parameters for both machines.

### 2.2. Image Processing

#### 2.2.1. Pre-Processing

**Figure 1:**
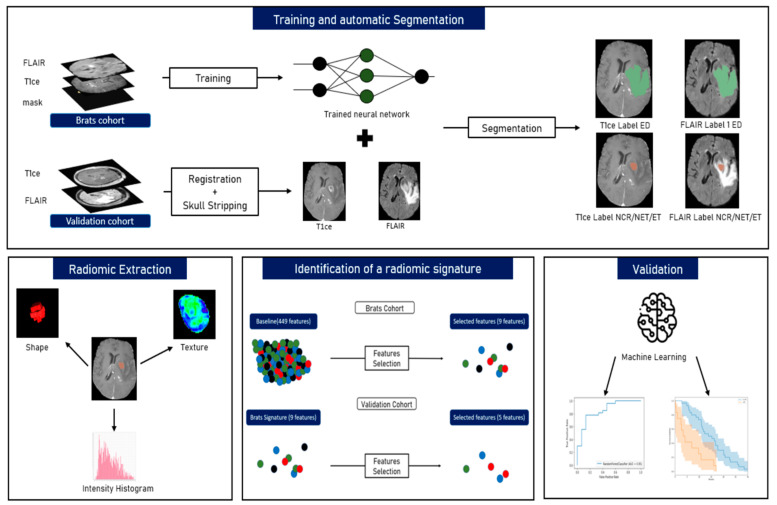
The workflow of radiomics and analysis used in this study. The overall procedure of identifying an MRI radiomics signature model and a practical ML model for the stratification of the GBM patients’ prognosis based on OS.

The whole pre-processing and post-processing pipelines are summarized in Figure 1. As the BraTS cohort was already pre-processed (pixel-wise normalization, co-registration and skull-stripping), the same pre-processing pipeline was applied on the validation set thanks to the BraTS preprocessor from the BraTS toolkit [27]. First, the T2-FLAIR images were registered on the T1ce images using the Advanced normalization tools (ANTs) software. Then, T1ce images were registered on the T1-weighted BraTS atlas and the same transformation was applied on the previously registered T2-FLAIR images to have every imaging modality from both cohorts in the same spatial coordinates. Afterwards, the HD-BET brain extraction tool [28] was used on all images of the validation cohort for skull-stripping.

#### 2.2.2. Tumour Segmentation

The nn-Unet [29] framework was used to segment the tumours on the MR images of the validation cohort. This neural network ranked first in the 2020 BraTS challenge on an identical dataset as the 2019 challenge’s segmentation task. nn-Unet offers a pre-trained brain tumour segmentation model. However, it needs four MRI modalities for the creation of the 3-labeled mask, while our validation cohort had only T1ce and T2-FLAIR. Therefore, the use of the pre-trained inference model available on the GitHub repository of the authors was not possible. Consequently, a new training task was carried out on the BraTS cohort using only these two modalities. The architecture of the neural network was kept untouched. This newly trained nn-Unet model was then applied for the segmentation of the validation cohort. All the segmentations were verified by a trained radiologist (AS with more than 10 years of experience). Eventually, the NCR/NET and ET regions were gathered within the same label in every mask.

#### 2.2.3. Radiomics and Features Extraction Technique

On both cohorts, radiomics were extracted from the two sub-masks (NCR/NET/ET and ED) on the T1ce and T2-FLAIR MRI modalities using the Python library Pyradiomics [30]. The sub-masks from the BraTS ground-truth were utilized for the BraTS cohort while the radiomics extraction of the validation cohort was performed on the images automatically segmented by the nn-Unet model. The radiomic set of features included: 18 first-order statistics, 14 shape-based features, 24 grey level co-occurrence matrix features (GLCM, texture), 16 grey level run length matrix features (GLRLM, texture), 16 grey level size zone matrix features (GLSZM, texture), 5 original neighboring grey tone difference matrix and 14 grey level dependence matrix features (GLDM, texture), 5 neighbouring grey tone Difference Matrix (NGTDM, texture). In the end, 448 radiomics (112 from each sub-mask and modality) were analyzed in addition to the age of the patients.

### 2.3. Machine Learning (ML) Algorithms

#### 2.3.1. BraTS Cohort Survival Analysis

Several ML algorithms were evaluated to process the BraTS cohort along 3 binary classification tasks: surviving less or more than 9, 12, and 15 months, respectively. GBM patients of the BraTS cohort were divided into train and test datasets. The proportion of each class in every classification model was kept in the train and test datasets. Cross-validations (CV = 5) were applied on the train datasets to avoid overfitting and to find the best feature-scaling method, classifier and hyper-parameter. Then, the best ML model found was trained on the overall train-set and evaluated on the test-set. Using the Scikit-learn Python library, 3 scaling methods (standard scaler, min–max scaler and robust scaler) and 7 classifiers (KNN, random forest, logistic regression gradient boosting, AdaBoost, naïve Bayes and SVM) were tested. Performances on the cross-validations were assessed using mean AUC over the 5 folds. In addition, due to the high dimensionality of the data, a feature selection using only the train-sets was imposed (from every fold of the cross-validation and the final evaluation) to be then validated on the test-sets. Only features with a concordance-index (c-index) with an OS superior to the threshold were kept. Afterwards, the correlation between all the remaining variables was measured; if it was superior to a second threshold, the one with the lowest c-index was removed. The two threshold values were estimated by cross-validation. Due to the imbalanced classes in the 9- and 15-month models (Table 3), a resampling method, namely the random over sampler tool from imblearn Python package, was used to avoid the overfitting of the majority class.

#### 2.3.2. Identification of Radiomic Signature

To validate a potential radiomic signature for the survival prediction of GBM patients, the features selected on the BraTS cohort were used to classify the patients of the validation cohort into two groups: survival < or > 50th percentile in terms of OS (median of 22 months). Only the features selected in at least two models trained on the BraTS cohort were retained. Then, the same data processing was applied to the validation cohort (separation in train and test-set, cross-validation, training, and validation). The c-index and correlation thresholds were retained to select the most appropriate signature.

### 2.4. Statistical Analysis

OS was calculated as the number of days between the diagnosis of GBM and death. Concordance index (C-index) [31] was used as an evaluation metric and for the selection of features. The area under the curve (AUC) of the receiving operating characteristic (ROC) curve was used to evaluate the classification performance. Kaplan–Meier curves and log-rank statistics served to assert whether or not the binary classification models distinguish two significantly different populations in terms of OS.

## 3. Results

### 3.1. OS Outcomes on BraTS Cohort

Several ML algorithms were tested for each model. On the cross-validation, an RFT (random forest) obtained the best score for the 9- and 12-months classification models with a mean AUC of 0.75 ± 0.04 (±1 standard deviation) and 0.74 ± 0.14, respectively. For the 15-months model, a logistic regression had a mean AUC value of 0.69 ± 0.09. These best models on the cross-validations had an AUC on the test-sets of 0.85 (0.92 on the train-set) for the 9-months model, 0.74 (0.88) for 12-months and 0.58 (0.75) for 15 months. Precision and recall for each class of each classification model are provided in Table 4.

**Figure 2:**
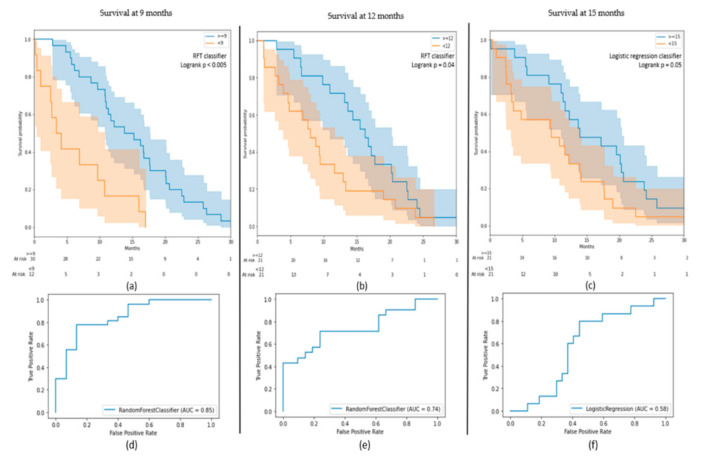
(**a**–**c**) Kaplan–Meier curves of the results on the test-sets for the 9-, 12-, and 15-months models; (**d**–**f**) receiver operating characteristic (ROC) curves of the results on the test-sets for the 9-, 12-, and 15-months models.

Although the 15-months model had a low AUC value, it has succeeded to stratify GBM patients into two significantly different populations on the test-set (log-rank *p* = 0.05) with well-separated Kaplan–Meier curves (Figure 2). The survival probability at 15 months is equal to 0.24 for the subgroup “before 15 months” and 0.48 for “beyond 15 months”. The two other models also had two significantly different populations on the test sets (log-rank *p* = 0.04 for 12 months and log-rank *p* < 0.005 for 9 months). The class “before 12 months” had a survival probability of 0.29 at 12 months while the class “beyond 12 months” had a probability of 0.71. The results on the 9-months model were more significant with survival probabilities of 0.33 and 0.77 for “before 9 months” and “beyond 9 months”, respectively. These probabilities went down to 0 versus 0.37 for the same classes.

### 3.2. Radiomic Signature

**Figure 3:**
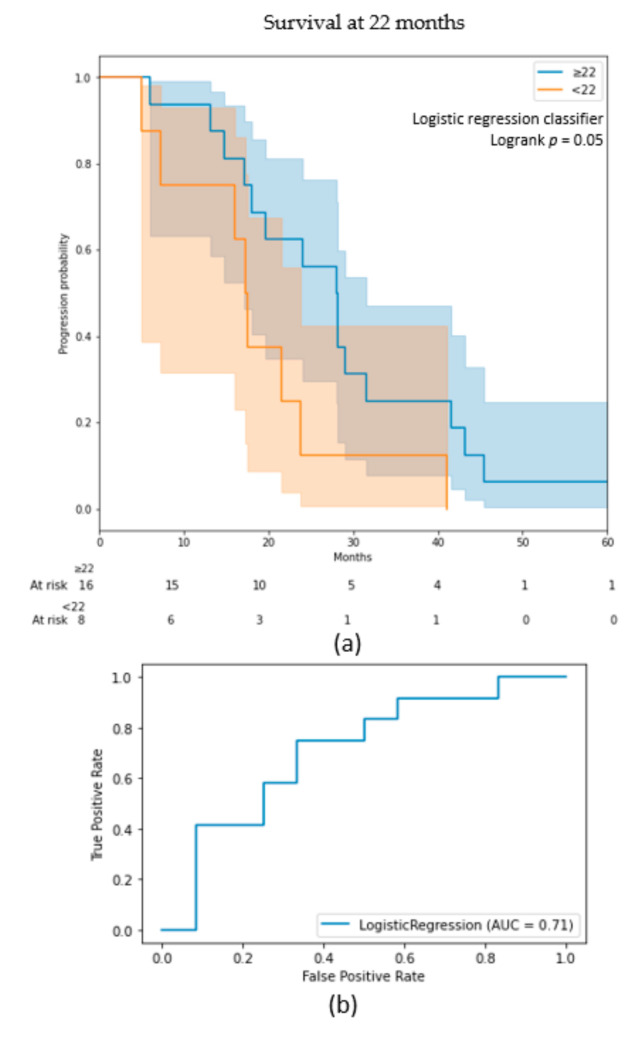
(**a**) Kaplan–Meier curve of the results on the test-set for the 22-months model; (**b**) receiver operating characteristic (ROC) curve of the results on the test-set for 22-months model.

The three classification models on the BraTS cohort selected 14, 13, and 9 features for the 9-, -12, and 15-months models, respectively. Six features were chosen in each model: age, one shape radiomic from ED’s T1ce sub-mask, one shape and one texture radiomics from NCR/NET/ET’s T1ce sub-mask, and one histogram intensity with one texture radiomics from NCR/NET/ET’s FLAIR sub-mask. Three other features were present in two models over the three: one shape radiomic from ED’s FLAIR sub-mask and two texture radiomics from NCT/NET/ET’s FLAIR sub-mask. These nine features were then used for the prediction of patients surviving less or more than the OS median of 22 months in the validation cohort. On this cohort, a logistic regression insured the best performance on the cross-validation with a mean AUC of 0.60 ± 0.10. This result was obtained due to a small c-index threshold of 0.51 in the feature selection. The algorithm reached an AUC of 0.71 on the test set (0.65 on the train). The Kaplan–Meier curve (Figure 3) showed two well-separated curves which represent two significantly different populations (log-rank *p* = 0.05). Among the nine pre-selected features, five were kept in the final model of the validation cohort. The age, sphericity, and the NCT/NET/ET sub-mask appear to be of utmost importance in the prediction of OS within both our cohorts (Table 5). Models using the age as only feature were also trained. The age-only factor models had every time lower results than the radiomic and age models. Indeed, it obtained AUC’s on the test-sets equal to 0.69, 0.69, 0.62 and 0.47 for the 9-, 12-, 15- and 22-months models. Only the 9-months model succeeded to significantly stratify two different population. The Kaplan–Meier curves of 9-, 12-, 15- months models (Figure S1) and 22- months model (Figure S2) are available in the Supplementary Materials (Figures S1 and S2).

## 4. Discussion

In this study, ML algorithms based on radiomics from MR imaging were designed for the survival prediction of GBM patients. A total of 448 radiomics from T1ce and FLAIR images were extracted from two independent cohorts of GBM patients. A radiomic signature was identified on the first cohort of 210 patients, arisen from the train dataset of the 2019 BraTS challenge. Then, the robustness of this signature for survival prediction of GBM was tested on a validation cohort composed of 116 patients. The results of this study suggest that these models can provide some valuable insights into the OS of GBM patients. The novel classification models successfully stratified patients into two significantly distinct populations, thus suggesting a possible role in the identification of GBM phenotypes. This classification reflects the aggressiveness of some phenotypes and might permit an optimal selection of therapy with personalized management based on the patient and tumor characteristics.

Survival analysis was performed at three different endpoints: 9, 12, and 15 months on the 2019 BraTS challenge. Based on the features selection, a radiomic signature of nine features were identified including eight radiomics in addition to age. Using ML algorithms, the signature successfully stratified two unique populations at every endpoint. For instance, Chen et al. [22] reported a radiomic signature of four features for the stratification of GBM patients with the survival of more or less than 12 months using a Cox proportional hazard regression model. Their model obtained similar AUC’s as our models. However, their signature was only composed of texture radiomics that were from the same sub-type as the contrast radiomic of our signature (grey level co-occurrence matrix features). They did not select any shape radiomic for example. These differences could be partially explained by the fact they used minimum redundancy feature selection method, while we used a feature selection based on the C-index. Similarly, Kickingereder et al. [17] used an eight-radiomics-based model to successfully classify a cohort of GBM patients into low-, medium- and high-risk groups, still using a multivariate Cox model. Their signature was composed of six texture radiomics (only the entropy was common to the Chen et al.’s signature while none of them was found in our study) and two shape radiomics such as sphericity. Strength and sphericity were also important parameters in our model. These features were among those selected for the successful prediction of outcomes in GBM patients treated with bevacizumab in a previous publication [32]. Adding age as a clinical variable, which also appeared in several reported survival models, appeared to be highly relevant in both our cohorts. Moreover, as stressed by our age-only factor models, radiomics significantly improve the OS prediction. Kickingereder et al. also found that hybrid models using clinical variables along radiomics should significantly improve the results compare to only-radiomics or only-clinical models [17]. Even though several reported radiomic signatures have successfully stratified GBM patients based on OS, none were able to validate their signature on a second independent cohort, to the best of our knowledge. This last step of validation is crucial for the assertion of a radiomic signature and its generalization, as the radiomic signature can vary from one to another, even though the subtypes can be similar. Indeed, one of the main limitations with the use of ML algorithms based on radiomics is the lack of reproducibility [33]; in this study, selected features were relevant, reproducible, and robust in two distinct populations. However, even though the BraTS cohort was multicentric, further validation on a new independent cohort was still needed to ensure the generalization and the robustness of our radiomic signature.

The new 2021 World Health Organization (WHO) classification of central nervous system tumors recognizes molecular evaluation as a critical step in their classification. Mutation status of the IDH1 (isocitrate dehydrogenase), IDH2, and the co-deletion of 1p/19q are crucial for diagnosis, prognosis and prediction of chemotherapy benefit [34]. Despite the recent advances in the molecular and genomic understanding of GBM, MGMT methylation status remains the most studied predictive biomarker of GBM, which is predictive of an improved response to alkylating chemotherapy such as temozolomide [35]. On the other hand, the presence of IDH genes mutation has been identified as a positive prognostic marker and linked to a different clinical outcome compared to *IDH* wild-type (WT) GBM [36]. To further improve the prognostic and predictive potential of non-invasive biomarkers, imaging models, particularly with the advent of radiomic phenotyping based on multi-parametric MRI data, might improve survival predictions when integrated into the clinical and genetic status of GBM patients with glioblastoma [37]. Future building of predictive models for survival could induce more accurate and robust models with the addition of further clinical and molecular features. For example, MGMT (O6-methylguanine–DNA methyltransferase) promoter methylation [7], transcriptomic [38], DCE-MRI [16], blood derive biomarkers [39] or diffusion MRI [40] have already been studied for their potential in the survival prediction of GBM patients.

One relatively major limitation of this paper is the difference in the median OS of the two populations (12 months in the BRATS cohort versus 22 months in the validation cohort). Nevertheless, the BRATS population was largely heterogeneous due to the multicentricity without relevant data on the initial management of patients and the absence of a homogenous therapeutic strategy among the different cancer centers. While on the other hand, the validation cohort included GBM patients from a single expert center with a standard protocol (Stupp protocol) on initial diagnosis as well as on relapsing setting (bevacizumab) in addition to an adapted supportive care program for GBM, thus explaining the vast difference in OS between the two cohorts. Nevertheless, this limitation might represent an actual strength for this study since adapting the same features and survival model from the BRATS population have induced successful outcomes in the validation cohort similar to the training population, despite having two distinct populations with different characteristics.

In conclusion, a signature of radiomics combined with age was identified which has successfully stratified a cohort of GBM patients into two distinct populations on 3 ML binary classification models for survival at 9,12 and 15 months. Additionally, this model significantly stratified two populations with different survival outcomes on a completely independent cohort of GBM patients. This study highlights the key role of radiomics for survival prediction and their potential of creating a daily clinical routine tool for decision-making and a more personalized approach.

## Figures and Tables

**Table 1:** Patient characteristics of the validation cohort.

Characteristics	Value
Age----------years	
Mean	59
Median	58.8
Min-max	26–80
Sex----------*n* (%)	
Female	44 (38%)
Male	72 (62%)
OS----------months	
Mean	27.33
Median	21.87
Min-max	3.93–117
Tumour location----------*n* (%)	
Left	58 (50%)
Right	58 (50%)
Multifocal	0 (0%)
Surgery----------*n* (%)	
Yes	81 (70%)
No	35 (30%)

**Table 2:** MRI parameters.

Machine	Weighting	Sequence	TR	TE	Slice Thickness
Optima MR450w 1.5 T *Installed in 2016, 70 cm tunnel, 32 channels, 40 cm z-axisFOV, gradient 40 mT/m SR 200 T/m/s.*	T1 pre-contrast	3D rapid gradient echo	9 ms	4.2 ms	1 mm
	T2-FLAIR	Turbo spin echo	7002 ms	138 ms	1.4 mm
	DWI	EPI, two b-values (0 and 1000 mm/s)	3349 ms	79 ms	4 mm
	T1 post-contrast	3D rapid gradient echo	6.1 ms	1.2 ms	1 mm
Discovery MR 750w 3 T *Installed in 2012, 70 cm tunnel, 32 channels, 50 cm z-axisFOV, gradient 44 mT/m SR 200 T/m/s.*	T1 pre-contrast	3D rapid gradient echo	9 ms	2.1 ms	1 mm
	T2-FLAIR	Turbo spin echo	7002 ms	118 ms	1 mm
	DWI	EPI, two b-values (0 and 1000 mm/s)	3349 ms	62.6 ms	3 mm
	T1 post-contrast	3D rapid gradient echo	6.1 ms	2.1 ms	1 mm

**Table 3:** Classes’ distribution before resampling.

Model	Classes	Number (%)
9 months survival	<9	73 (35%)
	≥9	137 (65%)
12 months survival	<12	105 (50%)
	≥12	105 (50%)
15 months survival	<15	136 (68%)
	≥15	74 (32%)

**Table 4:** Results metrics of the classification models on the test sets.

Model	Cohort	Best Classifier	AUC on Test (on Train)	Classes	Precision	Recall
9 months survival	BraTS	RFT	0.85 (0.92)	≥9	0.77	0.85
				<9	0.67	0.53
12 months survival	BraTS	RFT	0.74 (0.88)	≥12	0.71	0.71
				<12	0.71	0.71
15 months survival	BraTS	Logistic regression	0.58 (0.75)	≥15	0.48	0.67
				<15	0.76	0.59
22 months survival	Validation	Logistic regression	0.71 (0.65)	≥22 <22	0.62 0.75	0.83 0.50

**Table 5:** Essential features found in the feature selection based on their C-index with the OS on the train sets.

Feature	Source	Sub-Mask	Radiomic Type	9 Months (C-index)	12 Months (C-index)	15 Months (C-index)	22 Months (C-index)	Correlation/Anticorrelation with OS
Sphericity	Gadolinium	ED	Shape	0.59	0.58	0.59	-	Correlation
Sphericity	Gadolinium	NCT/NET/ET	Shape	0.58	0.56	0.56	0.53	Correlation
Minimum	Flair	NCT/NET/ET	Histogram intensity	0.59	0.59	0.59	-	Correlation
Strength	Flair	NCT/NET/ET	Texture	0.57	0.58	0.58	0.57	Correlation
Strength	Gadolinium	NCT/NET/ET	Texture	0.56	0.57	0.58	-	Correlation
Age	Flair	-	-	0.63	0.64	0.62	0.53	Anticorrelation
Major axis length	Flair	ED	Shape	-	0.58	0.56	-	Anticorrelation
Contrast	Flair	NCT/NET/ET	Texture	0.56	0.56	-	0.58	Correlation
Coarseness	Flair	NCT/NET/ET	Texture	0.59	-	0.60	0.53	Correlation

## Data Availability

The data presented in this study are available on request from the corresponding author. The data are not publicly available due to privacy and ethical concerns.

## Supplementary Materials

The following are available online at https://www.mdpi.com/article/10.3390/diagnostics11112043/s1, Figure S1. (a–c) Kaplan–Meier curves of the results on the test-sets for the 9-, 12-, and 15-month models using the age as the only feature; (d–f) receiver operating characteristic (ROC) curves of the results on the test-sets for the 9-, 12-, and 15-months models using the age as the only feature. Figure S2. (a) Kaplan–Meier curve of the results on the test-set for the 22-months model using the age as the only feature; (b) receiver operating characteristic (ROC) curve of the results on the test-set for 22-months model using the age as the only feature.

## References

[1] Dolecek T.A., Propp J.M., Stroup N.E., Kruchko C. (2012). CBTRUS Statistical Report: Primary Brain and Central Nervous System Tumors Diagnosed in the United States in 2005–2009. Neuro Oncol..

[2] Nam J.Y., de Groot J.F. (2017). Treatment of Glioblastoma. J. Oncol. Pract..

[3] Wick W., Osswald M., Wick A., Winkler F. (2018). Treatment of Glioblastoma in Adults. Ther. Adv. Neurol. Disord..

[4] Paolillo M., Boselli C., Schinelli S. (2018). Glioblastoma under Siege: An Overview of Current Therapeutic Strategies. Brain Sci..

[5] Wen P.Y., Kesari S. (2008). Malignant Gliomas in Adults. N. Engl. J. Med..

[6] Lapointe S., Perry A., Butowski N.A. (2018). Primary Brain Tumours in Adults. Lancet.

[7] Binabaj M.M., Bahrami A., ShahidSales S., Joodi M., Joudi Mashhad M., Hassanian S.M., Anvari K., Avan A. (2018). The Prognostic Value of MGMT Promoter Methylation in Glioblastoma: A Meta-analysis of Clinical Trials. J. Cell Physiol..

[8] Sottoriva A., Spiteri I., Piccirillo S.G.M., Touloumis A., Collins V.P., Marioni J.C., Curtis C., Watts C., Tavaré S. (2013). Intratumor Heterogeneity in Human Glioblastoma Reflects Cancer Evolutionary Dynamics. Proc. Natl. Acad. Sci. USA.

[9] Snuderl M., Fazlollahi L., Le L.P., Nitta M., Zhelyazkova B.H., Davidson C.J., Akhavanfard S., Cahill D.P., Aldape K.D., Betensky R.A. (2011). Mosaic Amplification of Multiple Receptor Tyrosine Kinase Genes in Glioblastoma. Cancer Cell.

[10] Francis J.M., Zhang C.-Z., Maire C.L., Jung J., Manzo V.E., Adalsteinsson V.A., Homer H., Haidar S., Blumenstiel B., Pedamallu C.S. (2014). EGFR Variant Heterogeneity in Glioblastoma Resolved through Single-Nucleus Sequencing. Cancer Discov..

[11] Kim J., Lee I.-H., Cho H.J., Park C.-K., Jung Y.-S., Kim Y., Nam S.H., Kim B.S., Johnson M.D., Kong D.-S. (2015). Spatiotemporal Evolution of the Primary Glioblastoma Genome. Cancer Cell.

[12] Hricak H., Abdel-Wahab M., Atun R., Lette M.M., Paez D., Brink J.A., Donoso-Bach L., Frija G., Hierath M., Holmberg O. (2021). Medical Imaging and Nuclear Medicine: A Lancet Oncology Commission. Lancet Oncol..

[13] Lohmann P., Galldiks N., Kocher M., Heinzel A., Filss C.P., Stegmayr C., Mottaghy F.M., Fink G.R., Jon Shah N., Langen K.-J. (2021). Radiomics in Neuro-Oncology: Basics, Workflow, and Applications. Methods.

[14] Riesterer O., Milas L., Ang K.K. (2007). Use of Molecular Biomarkers for Predicting the Response to Radiotherapy with or without Chemotherapy. J. Clin. Oncol..

[15] Liu Z., Wang S., Dong D., Wei J., Fang C., Zhou X., Sun K., Li L., Li B., Wang M. (2019). The Applications of Radiomics in Precision Diagnosis and Treatment of Oncology: Opportunities and Challenges. Theranostics.

[16] Pak E., Choi K.S., Choi S.H., Park C.-K., Kim T.M., Park S.-H., Lee J.H., Lee S.-T., Hwang I., Yoo R.-E. (2021). Prediction of Prognosis in Glioblastoma Using Radiomics Features of Dynamic Contrast-Enhanced MRI. Korean J. Radiol..

[17] Kickingereder P., Neuberger U., Bonekamp D., Piechotta P.L., Götz M., Wick A., Sill M., Kratz A., Shinohara R.T., Jones D.T.W. (2018). Radiomic Subtyping Improves Disease Stratification beyond Key Molecular, Clinical, and Standard Imaging Characteristics in Patients with Glioblastoma. Neuro Oncol..

[18] Sanghani P., Ang B.T., King N.K.K., Ren H. (2018). Overall Survival Prediction in Glioblastoma Multiforme Patients from Volumetric, Shape and Texture Features Using Machine Learning. Surg. Oncol..

[19] Baid U., Rane S.U., Talbar S., Gupta S., Thakur M.H., Moiyadi A., Mahajan A. (2020). Overall Survival Prediction in Glioblastoma With Radiomic Features Using Machine Learning. Front. Comput. Neurosci..

[20] Osman A.F.I. (2019). A Multi-Parametric MRI-Based Radiomics Signature and a Practical ML Model for Stratifying Glioblastoma Patients Based on Survival Toward Precision Oncology. Front. Comput. Neurosci..

[21] Macyszyn L., Akbari H., Pisapia J.M., Da X., Attiah M., Pigrish V., Bi Y., Pal S., Davuluri R.V., Roccograndi L. (2016). Imaging Patterns Predict Patient Survival and Molecular Subtype in Glioblastoma via Machine Learning Techniques. Neuro Oncol..

[22] Chen X., Fang M., Dong D., Liu L., Xu X., Wei X., Jiang X., Qin L., Liu Z. (2019). Development and Validation of a MRI-Based Radiomics Prognostic Classifier in Patients with Primary Glioblastoma Multiforme. Acad. Radiol..

[23] Menze B.H., Jakab A., Bauer S., Kalpathy-Cramer J., Farahani K., Kirby J., Burren Y., Porz N., Slotboom J., Wiest R. (2015). The Multimodal Brain Tumor Image Segmentation Benchmark (BRATS). IEEE Trans. Med. Imaging.

[24] Bakas S., Akbari H., Sotiras A., Bilello M., Rozycki M., Kirby J.S., Freymann J.B., Farahani K., Davatzikos C. (2017). Advancing The Cancer Genome Atlas Glioma MRI Collections with Expert Segmentation Labels and Radiomic Features. Sci. Data.

[25] Bakas S., Reyes M., Jakab A., Bauer S., Rempfler M., Crimi A., Shinohara R.T., Berger C., Ha S.M., Rozycki M. (2018). Identifying the Best Machine Learning Algorithms for Brain Tumor Segmentation, Progression Assessment, and Overall Survival Prediction in the BRATS Challenge. arXiv Prepr..

[26] Gupta A., Dwivedi T. (2017). A Simplified Overview of World Health Organization Classification Update of Central Nervous System Tumors 2016. J. Neurosci. Rural. Pract..

[27] Kofler F., Berger C., Waldmannstetter D., Lipkova J., Ezhov I., Tetteh G., Kirschke J., Zimmer C., Wiestler B., Menze B.H. (2020). BraTS Toolkit: Translating BraTS Brain Tumor Segmentation Algorithms into Clinical and Scientific Practice. Front. Neurosci..

[28] Isensee F., Schell M., Pflueger I., Brugnara G., Bonekamp D., Neuberger U., Wick A., Schlemmer H., Heiland S., Wick W. (2019). Automated Brain Extraction of Multisequence MRI Using Artificial Neural Networks. Hum. Brain Mapp..

[29] Isensee F., Jaeger P.F., Kohl S.A.A., Petersen J., Maier-Hein K.H. (2021). NnU-Net: A Self-Configuring Method for Deep Learning-Based Biomedical Image Segmentation. Nat. Methods.

[30] van Griethuysen J.J.M., Fedorov A., Parmar C., Hosny A., Aucoin N., Narayan V., Beets-Tan R.G.H., Fillion-Robin J.-C., Pieper S., Aerts H.J.W.L. (2017). Computational Radiomics System to Decode the Radiographic Phenotype. Cancer Res..

[31] Uno H., Cai T., Pencina M.J., D’Agostino R.B., Wei L.J. (2011). On the C-Statistics for Evaluating Overall Adequacy of Risk Prediction Procedures with Censored Survival Data. Stat. Med..

[32] Ammari S., Sallé de Chou R., Assi T., Touat M., Chouzenoux E., Quillent A., Limkin E., Dercle L., Hadchiti J., Elhaik M. (2021). Machine-Learning-Based Radiomics MRI Model for Survival Prediction of Recurrent Glioblastomas Treated with Bevacizumab. Diagnostics.

[33] Fournier L., Costaridou L., Bidaut L., Michoux N., Lecouvet F.E., de Geus-Oei L.-F., Boellaard R., Oprea-Lager D.E., Obuchowski N.A., Caroli A. (2021). European Society of Radiology. Correction to: Incorporating Radiomics into Clinical Trials: Expert Consensus Endorsed by the European Society of Radiology on Considerations for Data-Driven Compared to Biologically Driven Quantitative Biomarkers. Eur. Radiol..

[34] Louis D.N., Perry A., Wesseling P., Brat D.J., Cree I.A., Figarella-Branger D., Hawkins C., Ng H.K., Pfister S.M., Reifenberger G. (2021). The 2021 WHO Classification of Tumors of the Central Nervous System: A Summary. Neuro Oncol..

[35] Hegi M.E., Diserens A.-C., Gorlia T., Hamou M.-F., de Tribolet N., Weller M., Kros J.M., Hainfellner J.A., Mason W., Mariani L. (2005). MGMT Gene Silencing and Benefit from Temozolomide in Glioblastoma. N. Engl. J. Med..

[36] Dahlrot R.H., Kristensen B.W., Hjelmborg J., Herrstedt J., Hansen S. (2013). A Population-Based Study of Low-Grade Gliomas and Mutated Isocitrate Dehydrogenase 1 (IDH1). J. Neurooncol..

[37] Bae S., Choi Y.S., Ahn S.S., Chang J.H., Kang S.-G., Kim E.H., Kim S.H., Lee S.-K. (2018). Radiomic MRI Phenotyping of Glioblastoma: Improving Survival Prediction. Radiology.

[38] Sorokin M., Raevskiy M., Zottel A., Šamec N., Vidmar M.S., Matjašič A., Zupan A., Mlakar J., Suntsova M., Kuzmin D.V. (2021). Large-Scale Transcriptomics-Driven Approach Revealed Overexpression of CRNDE as a Poor Survival Prognosis Biomarker in Glioblastoma. Cancers.

[39] van Linde M.E., van der Mijn J.C., Pham T.V., Knol J.C., Wedekind L.E., Hovinga K.E., Aliaga E.S., Buter J., Jimenez C.R., Reijneveld J.C. (2016). Evaluation of Potential Circulating Biomarkers for Prediction of Response to Chemoradiation in Patients with Glioblastoma. J. Neurooncol..

[40] Brancato V., Nuzzo S., Tramontano L., Condorelli G., Salvatore M., Cavaliere C. (2020). Predicting Survival in Glioblastoma Patients Using Diffusion MR Imaging Metrics—A Systematic Review. Cancers.

