# Supplementary material for: A Predictive Clinical-Radiomics Nomogram for Survival Prediction of Glioblastoma Using MRI
Source: Diagnostics (Basel). 2021 Nov 4;11(11):2043. doi: 10.3390/diagnostics11112043 (PMC8624566; doi:10.3390/diagnostics11112043)
Supplement: Supplementary file 1 [file diagnostics-11-02043-s001.zip › diagnostics-1416775-supplementary.pdf]

Supplementary material

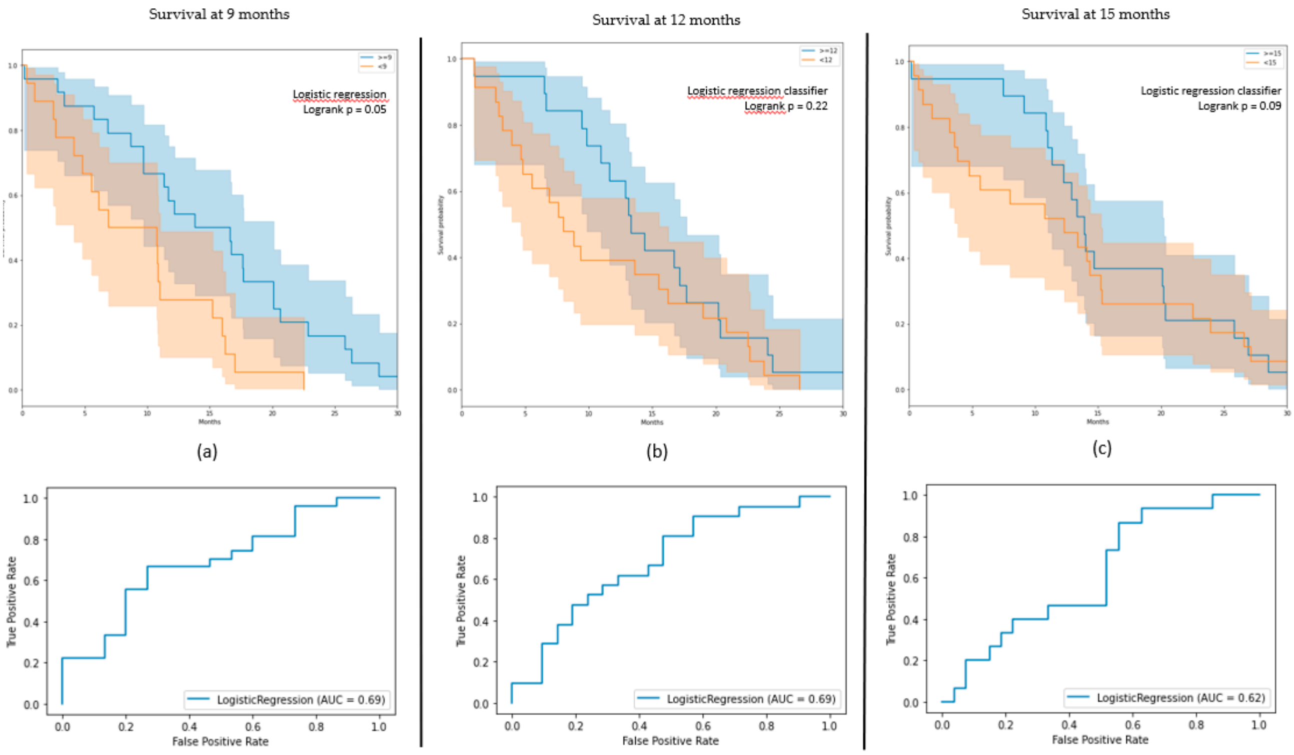

Figure S1. (**a**–**c**) Kaplan–Meier curves of the results on the test-sets for the 9-, 12-, and 15-month models using the age as the only feature; (**d**–**f**) receiver operating characteristic (ROC) curves of the results on the test-sets for the 9-, 12-, and 15-months models using the age as the only feature.

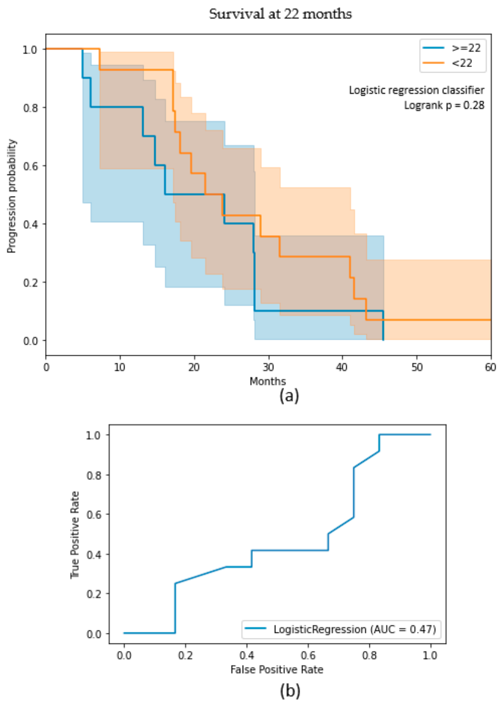

Figure S2. (**a**) Kaplan–Meier curve of the results on the test-set for the 22-months model using the age as the only feature; (**b**) receiver operating characteristic (ROC) curve of the results on the test-set for 22-months model using the age as the only feature.
